# Proteomic Signature of Extracellular Vesicles for Lung Cancer Recognition

**DOI:** 10.3390/molecules26206145

**Published:** 2021-10-12

**Authors:** Svetlana E. Novikova, Natalia A. Soloveva, Tatiana E. Farafonova, Olga V. Tikhonova, Pao-Chi Liao, Victor G. Zgoda

**Affiliations:** 1 Orekhovich Institute of Biomedical Chemistry, Russian Academy of Medical Sciences, Pogodinskaya 10, 119121 Moscow, Russia; novikova.s.e3101@gmail.com (S.E.N.); farafonova.tatiana@gmail.com (T.E.F.); tiolika@gmail.com (O.V.T.); 2 Department of Environmental and Occupational Health, College of Medicine, National Cheng-Kung University, 1 Dasyue Rd., East District, Tainan 701, Taiwan; liaopc@mail.ncku.edu.tw

**Keywords:** extracellular vesicles, proteomic signature, mass spectrometry, lung cancer, SRM, stable isotope-labeled peptide standards

## Abstract

The proteins of extracellular vesicles (EVs) that originate from tumors reflect the producer cells’ proteomes and can be detected in biological fluids. Thus, EVs provide proteomic signatures that are of great interest for screening and predictive cancer diagnostics. By applying targeted mass spectrometry with stable isotope-labeled peptide standards, we assessed the levels of 28 EV-associated proteins, including the conventional exosome markers CD9, CD63, CD81, CD82, and HSPA8, in vesicles derived from the lung cancer cell lines NCI-H23 and A549. Furthermore, we evaluated the detectability of these proteins and their abundance in plasma samples from 34 lung cancer patients and 23 healthy volunteers. The abundance of TLN1, TUBA4A, HSPA8, ITGB3, TSG101, and PACSIN2 in the plasma of lung cancer patients was measured using targeted mass spectrometry and compared to that in plasma from healthy volunteers. The most diagnostically potent markers were TLN1 (AUC, 0.95), TUBA4A (AUC, 0.91), and HSPA8 (AUC, 0.88). The obtained EV proteomic signature allowed us to distinguish between the lung adenocarcinoma and squamous cell carcinoma histological types. The proteomic cargo of the extracellular vesicles represents a promising source of potential biomarkers.

## 1. Introduction

According to the World Health Organization, lung cancer (LC) is the most common cause of cancer-related death (1.80 million cases). It also ranks second in terms of occurrence (https://www.who.int/news-room/fact-sheets/detail/cancer, accessed on 6 October 2021).

LC includes two main histological classes: small-cell lung cancers (SCLCs) (10–15% of all LC cases), originating from hormonal cells in the lung, and non-small-cell lung carcinomas (NSCLCs) (90–85% of all LC cases), derived from the epithelium of the bronchi and alveoli [1]. Furthermore, NSCLC is divided into squamous cell carcinoma (25–30% of LC cases), adenocarcinoma (40% of LC cases), and large-cell carcinoma (10% of LC cases). The international TNM-based staging system describes the extent of the disease in terms of the primary tumor size (T), involvement of regional lymph nodes (N), and distant metastatic spread (M). According to the histological class and the TNM subset, NSCLC tumors are assigned to one of four stages (I–IV), and the appropriate treatment is determined.

LC is often diagnosed at a late stage (in up to 70% of cases) due to its non-specific symptoms, e.g., cough, chest pain, and dyspnea. The prognosis for patients diagnosed at a late stage is unfavorable, with a five-year survival rate of 3.9% [2]. Thus, screening methods for LC, especially at the early stage when the tumor is resectable, are urgently needed. To date, imaging techniques such as low-dose computed tomography (LDCT) are available. The application of LDCT led to a reduction in mortality in patients from a high-risk group with a history of smoking [3]. The high false-positive rate (up to 23%) and over-diagnosis are the main disadvantages of the LDCT method [4]. To avoid unnecessary CT examinations and related radiotherapy burdens, additional or alternative diagnostic approaches should be implemented.

The promising concept of liquid biopsy aims to detect biomolecules (circulating tumor DNA (ctDNA), tumor-associated autoantibodies, and microRNA (miRNA)), cells (tumor-educated platelets and circulating tumor cells (CTCs)), and extracellular vesicles (EVs) that originate from malignant tumors and circulate in biological fluids [5]. EVs, which are classified into exosomes (30–100 nm in diameter) and microvesicles (50–1000 nm in diameter), contain DNA, RNA, and proteins encapsulated into the bilayer lipid membranes. An EV’s molecular cargo mirrors the content of the producer cancer cells, which makes it a valuable source of tumor-related biomarkers.

Recent advances in mass spectrometry allow for the detection and quantification of thousands of proteins in cells, tissues, and biological fluids. As a powerful analytical tool, mass spectrometric analysis has been used to unravel the fundamental role of EVs in cancer, e.g., the modulation of the tumor microenvironment, mediating immune evasion, and contributing to metastasis [6,7]. Mass-spectrometry-based proteomics is also useful for exploring EVs as a source of diagnostic makers, prognostic markers (severity of the outcome), and predictive markers (e.g., response to treatment).

In a recent study using high-resolution mass spectrometry, it was found that proteins such as extracellular matrix components, cell-adhesion proteins, complement factors, histones, proteasome subunits, and membrane transporters were upregulated in EVs originating from cisplatin-resistant A549 lung cancer cells, as compared to their wild-type counterparts [8]. On the basis of shotgun mass spectrometry followed by Western blot (WB) and ELISA assays, lipopolysaccharide-binding proteins (LBPs) allowed EVs derived from the blood of metastatic and non-metastatic NSCLC patients to be distinguished [9]. Label-free mass spectrometric quantification was performed on exosomes that were isolated from the plasma of patients with malignant and benign pulmonary nodules. The results indicated that the fibrinogen beta chain (FBB) and fibrinogen gamma chain (FGB) levels were significantly higher in the exosomes of the group with malignant nodules [10].

Targeted mass spectrometry or selected reaction monitoring (SRM) holds a special place among proteomic methods. As a result of its high sensitivity and selectivity, this method enables the detection of low-abundance proteins, e.g., transcription factors in complex biological matrices, such as blood plasma or cell lysates [11,12]. Moreover, SRM allows for the simultaneous absolute quantitative analysis of numerous proteins, which is of great importance for the quantitative assessment of diagnostically significant proteins [13]. Thus, the SRM technique may be considered as an alternative to immunochemical assays, which are conventionally used in clinical diagnostics.

Regarding LC, upon applying semi-quantitative SRM assays, retinal dehydrogenase 1 (AL1A1), 4F2hc cell-surface antigen heavy chain (4F2hc/CD98), apolipoprotein A-I (APOA), and β-enolase (ENOB) were found to be upregulated in formalin-fixed paraffin-embedded (FFPE) tissue samples from large-cell neuroendocrine lung carcinoma (LCNEC) patients compared to other lung cancer subtypes [14]. Moreover, napsin-A (NAPSA) and anterior gradient protein 2 homolog (AGR2) were proposed as stage-related proteins based on label-free SRM quantification in FFPE samples from stage IA and IIIA NSCLC patients [15]. The quantification of 42 PI3K–mTOR and MAPK phosphorylation sites was performed using different targeted proteomic techniques, including SRM, in NSCLC cell lines (PC9 and H1975), in order to unravel the mechanism of tyrosine kinase inhibitor resistance in LC [16]. However, there is a lack of data on EV-associated protein quantification using SRM with isotopically labeled peptide standards (SRM/SIS). SIS standards allow for the absolute quantification of targeted proteins and enable high-confidence detection due to co-elution and similar fragmentation patterns for SIS standards and their natural counterparts.

In a recent study, we performed label-free quantitative proteomic profiling of EVs originating from the lung cancer cell lines NCI-H23 and A549, as well as from various colon cancer cell lines. From the 651 EV proteins identified, we found 11 universal, 8 tissue-specific, and 29 line-specific markers, the levels of which were increased in EVs compared to whole-cell lysates [17]. 

In this study, we aimed to quantify the levels of 28 EV-associated proteins from the aforementioned study in NCI-H23 and A549 EVs as well as in the plasma of lung cancer patients and healthy volunteers using SRM/SIS. The data obtained using the plasma of healthy volunteers and LC patients allowed us to distinguish between healthy and cancer conditions. Moreover, we were able to distinguish between patients with lung adenocarcinoma and squamous cell carcinoma histological types.

## 2. Results

### 2.1. Verification of Protein Levels of 28 EV-Associated Markers in A549 and NCI-H23 Cell Lines by SRM Analysis

On the basis of proteomic profiling data for the EVs and whole-cell lysates (WhLs) of LC and colorectal cancer (CRC) cell lines obtained previously, we selected a set of 28 proteins whose levels were increased in EVs compared to the whole-cell lysates [17]. These EV proteins consisted of the following: conventional EV markers (CD82, CD81, CD9, CD63, and HSPA8), universal EV markers (CDC42, CNP, FN1, GNAI2, HSPG2, ITGB1, ITGB3, MVP, SLC2A1, TLN1, and TUBA4A), tissue-specific markers (LC-specific: EPS15, GPRC5A, and TSG101), and adenocarcinoma-cell-line-specific markers (A549 cell line: ITGA3, PACSIN2, PDCD6IP, PTGFRN, and RACGAP1; NCI-H23 cell-line-specific markers: ICAM1, MFGE8, and SDC4). For these, 36 proteotypic peptides, which were uniquely mapped onto the 28 EV-associated proteins above, were synthesized as stable isotope-labeled standards (SIS). All the peptides are listed in Supplementary Materials, Table S1 (“Peptides and proteins”).

The proteotypic peptides represent the “probes” for the measurement of the protein content in the sample. The efficiency of targeted mass spectrometric analysis largely depends on the specificity of the proteotypic peptide for the protein being measured. According to the Unicity checker tool (https://www.nextprot.org/tools/peptide-uniqueness-checker, accessed on 6 October 2021), all the peptides were uniquely specific for one protein-coding gene; among them, 14 peptides were uniquely mapped onto one protein isoform, while 14, 3, 2, and 3 peptides showed specificity for 2, 3, 4, and more than 5 isoforms of the same protein-coding gene, respectively (Figure S1a). Isoform specificity is an important factor when different isoforms involved in diverse biological processes need to be analyzed by SRM. The number of protein isoforms that could be measured by SIS per EV-associated protein is shown in Figure S1b. While the majority of prototypic peptides (28 peptides; 77% of all the peptides used for SRM analysis) were specific for one or two isoforms, the peptides STTPDITGYR and SYTITGLQPGTDYK, which were mapped onto FN1, corresponded to 15 isoforms of this protein (Figure S1b). 

**Figure 1:**
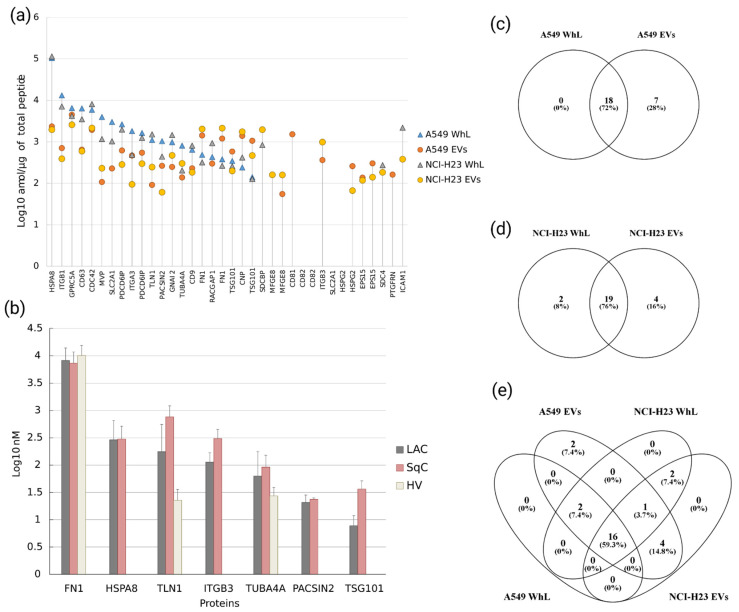
(**a**) Abundance of 34 peptides uniquely mapped onto 27 EV-associated proteins, which were measured in the EV and WhL samples obtained for A549 and NCI-H23 cell lines; Y-axis is log10 of the peptide content in amol/µg of total peptide; (**b**) abundance of 7 EV-associated proteins (FN1, HSPA8, TLN1, ITGB3, TUBA4A, PACSIN2, and TSG101) that were detected and measured in the blood plasma of 34 LC patients (23 and 11 patients with lung adenocarcinoma (LAC) and lung squamous cell carcinoma (SqC), respectively) and 23 healthy volunteers (HV); Y-axis is log10 of the protein concentration in nM; Venn diagrams demonstrate the overlap of the quantified proteins between EV and WhL of (**c**) the A549 LC cell line, (**d**) the NCI-H23 LC cell line, and (**e**) both cell lines studied.

By applying SRM/SIS, 34 of the 36 peptides, which were mapped onto 27 EV-associated proteins, were detected and measured in the EV and WhL samples obtained for the A549 and NCI-H23 cell lines (Figure 1a). The SRM signal for the peptides representing the conventional marker CD82 (SSFISVLQTSSSSLR and GEEDNSLSVR) was not detected. The detected protein abundances were in a range of almost three orders of magnitude. The protein HSPA8 was the most abundant in the WhL samples in both cell lines, with levels of 104.4 ± 22.8 fmol/µg (A549) and 114.4 ± 39.7 fmol/µg (NCI-H23) of the total sample protein content. The content of the EV-associated marker TSG101 was minimal in both cell lines, with levels of 0.140 ± 0.028 fmol/µg (A549) and 0.127 ± 0.026 fmol/µg (NCI-H23). Furthermore, in EV samples originating from both LC cell lines, retinoic-acid-induced protein 3 (GPRC5A) was the most abundant, with levels of 4.5 ± 0.05 fmol/µg (A549) and 2.5 ± 1.3 fmol/µg (NCI-H23). The lowest protein content was found for lactadherin (MFGE8) in A549-derived EV samples (0.05 ± 0.01 fmol/µg), and for basement-membrane-specific heparan sulfate proteoglycan core protein (HSPG2) in NCI-H23-derived EV samples (0.06 ± 0.07 fmol/µg). 

To measure the levels of eight proteins (CD82, EPS15, FN1, HSPG2, MFGE8, PDCD6IP, SLC2A1, and TSG101), we used two unique peptides per protein for the SRM analysis. It is considered that, the higher the coverage of the amino acid sequence achieved by the mass spectrometric analysis, the more reliable the detection of the corresponding protein. 

Figure 1c,d demonstrates that most of the proteins found in the WhL samples overlap with proteins detected in the EV samples, e.g., 18 and 19 proteins for the A549 and NCI-H23 cell lines, respectively, which illustrates the similarity of the vesicles and producer-cell molecular contents. Moreover, four proteins (MFGE8, ITGB3, HSPG2, and EPS15) were detected exclusively in the EV samples derived from both LC cell lines, and two proteins (CD81 and PTGFRN) were detected in EVs originating from the A549 cell line only (Figure 1e). 

### 2.2. SRM/SIS Analysis of EV-Associated Proteins in the Blood Plasma of LC Patients and Healthy Volunteers Reveals the Proteomic Signature of EVs

Using SRM/SIS, we quantified the EV-associated proteins in undepleted blood plasma obtained from 34 LC patients and 23 healthy volunteers. Patient information is shown in Table 1, and details are provided in Table S1 (“Patient Info”). 

There are several common methods for isolating EVs from blood plasma, e.g., using ultracentrifugation when the starting sample volume is 10–20 mL [18], applying a density gradient medium, or precipitation with polyglycols. In the latter case, a lower volume of blood plasma is required (approximately 0.1–1 mL) [19,20], but the EV isolation procedure can take a long time (overnight incubation is sometimes required) [21] and can be incompatible with mass spectrometry [18]. The large volume of blood required, laborious EV-isolation procedure, and additional step in sample handling make the routine analysis of EV-associated proteins difficult. The SRM method is distinguished by its high sensitivity and selectivity, which makes it possible to effectively analyze EV-associated proteins directly in whole-blood plasma samples taken with very small volumes (approximately 2.5 μL of plasma per SRM/SIS analysis). It should be noted that in this study we omitted EV isolation procedures for clinical samples and determined the content of EV-associated proteins directly in undepleted blood plasma, due to the advantages of the SRM/SIS method.

**Figure 2:**
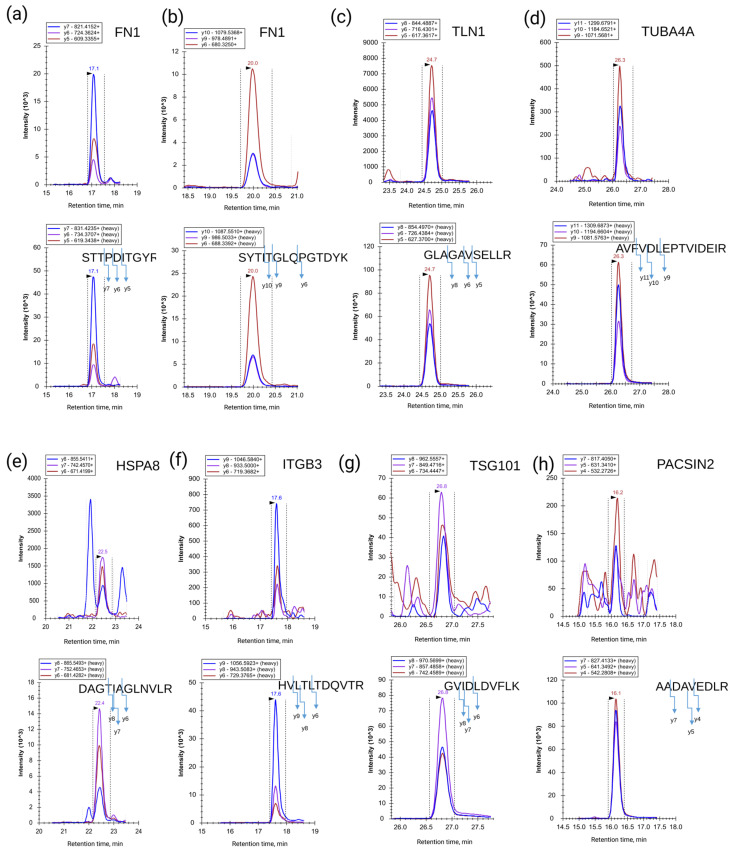
Trace of SRM transitions for native (upper panel) and stable isotope-labeled peptide standards (SISs) (lower panel) detected in blood plasma for EV-associated proteins: (**a**,**b**) fibronectin (FN1, peptides STTPDITGYR and SYTITGLQPGTDYK); (**c**) talin-1 (TLN1, peptide GLAGAVSELLR); (**d**) tubulin alpha-4A chain (TUBA4A, peptide AVFVDLEPTVIDEIR); (**e**) heat shock cognate 71 kDa protein (HSPA8, peptide DAGTIAGLNVLR); (**f**) integrin beta-3 (ITGB3, peptide HVLTLTDQVTR); (**g**) tumor susceptibility gene 101 protein (TSG101, peptide GVIDLDVFLK); and (**h**) protein kinase C and casein kinase substrate in neurons protein 2 (PACSIN2, peptide AADAVEDLR). These proteins were, together, denominated as an EV proteomic signature.

Overall, as a result of the SRM/SIS analysis, 7 out of 28 desired proteins were detected in the blood plasma of LC patients and healthy volunteers: fibronectin (FN1), talin-1 (TLN1), tubulin alpha-4A chain (TUBA4A), heat shock cognate 71 kDa protein (HSPA8), integrin beta-3 (ITGB3), tumor susceptibility gene 101 protein (TSG101), and protein kinase C/casein kinase substrate in neurons protein 2 (PACSIN2). The SRM traces for the detected peptides and the corresponding SIS standards are shown in Figure 2. The high-resolution spectra from the shotgun experiment are shown in Figures S3–S9. The proteins FN1, TLN1, TUBA4A, HSPA8, ITGB3, TSG101, and PACSIN2 were, together, denominated as an EV proteomic signature.

While fibronectin was detected in all the samples studied, PACSIN2 was only detected in nine LC (six LAC and three SqC) samples (Figure 2h). Fibronectin was also the most abundant EV-associated protein, with levels of 0.8 ± 0.3 and 1.1 ± 0.4 µM in the LC patient and healthy control blood plasma, respectively. Furthermore, TSG101 and PACSIN2 were only detected in LC patient blood plasma at low levels, i.e., 1.6 ±1.5 and 2.2 ± 1.5 nM, respectively. Thus, the EV proteomic signature covers a dynamic range of almost three orders of magnitude (Figure 1b).

**Figure 3:**
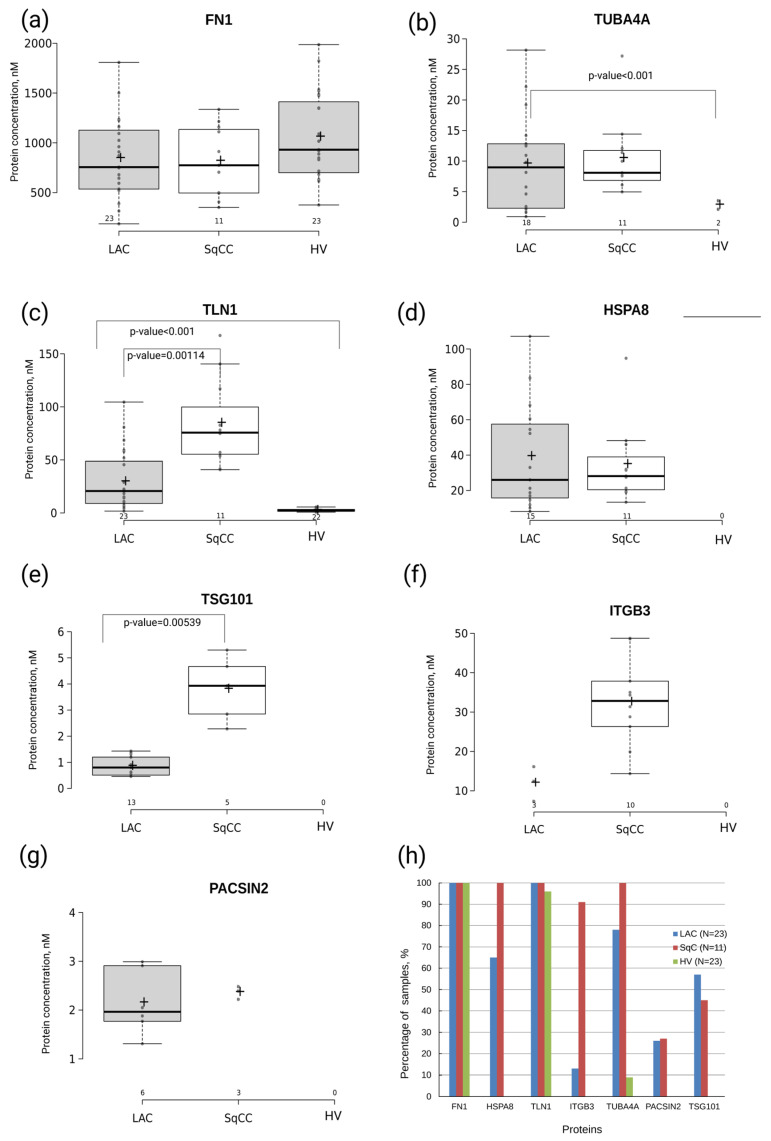
Results of the SRM/SIS measurements of the EV proteomic signature: (**a**) FN1; (**b**) TUBA4A; (**c**) TLN1; (**d**) HSPA8; (**e**) TSG101; (**f**) ITGB3; and (**g**) PACSIN2 in undepleted blood plasma derived from patients with lung adenocarcinoma (LAC, N = 23), lung squamous cell carcinoma (SqC, N = 11), and healthy volunteers (HV, N = 23). The distribution of protein content is shown as a boxplot chart if protein was measured in at least 5 samples per group. Protein concentration is shown in nM. The cross indicates the mean concentration of the detected proteins. (**h**) Percentage of sample in which EV-associated proteins were detected.

Fibronectin was detected by SRM with two peptide standards in all the samples studied. The observed contents of two proteotypic peptides, STTPDITGYR and SYTITGLQPGTDYK, for FN1 correlated with R^2^ = 0.95 (Figure S2a). The calculated average values of the peptide concentrations were considered to be the FN1 abundance in the sample. We did not observe significant differences in the FN1 abundances when comparing the samples from healthy volunteers (1.1 ± 0.4 µM) to LC patients (0.8 ± 0.3 µM) (Figure 3a).

Talin-1 was also detected in all the LC patient samples (N = 34) and in 22 out of 23 samples from healthy volunteers. The abundance of TLN1 was significantly higher (*t*-test *p*-value < 0.001) in the blood of LC patients (47 ± 41 nM) than in healthy controls (2.5 ± 1.1 nM) (Figure 3c). Moreover, TLN1 levels were almost three times higher (*p*-value = 0.00114) in patients with lung squamous cell carcinoma (SqC) (84.4 ± 27.4 nM) than in lung adenocarcinoma (LAC) patients (29.5 ± 12.2 nM) (Figure 3b). 

The protein TUBA4A was measured in 18 SqC patient samples (10.4 ± 4.2 nM) and 11 LAC patient samples (9.5 ± 3.9 nM), and was detected in two samples from healthy volunteers (2.8 ± 1.0 nM). Distinctive differences were observed between LC patients and healthy controls (HC) in terms of both the frequency of TUBA4A detection (29 out of 34 LC vs. 2 out of 23 HC) and protein levels (3.5-fold change; *p*-value < 0.001) (Figure 3c).

The proteins HSPA8, TSG101, ITGB3, and PACSIN2 were only detected and measured in LC patient samples. The content of HSPA8 was approximately the same for the two histological lung cancer subtypes (39.1 ± 16.8 nM in LAC samples (N = 15) vs. 34.6 ± 15.2 nM in SqC samples (N = 11)). The abundance of TSG101, which was detected in 13 LAC samples (0.8 ± 0.2 nM) and five SqC samples (3.8 ± 1.5 nM), was significantly higher for lung squamous cell carcinoma (*p*-value = 0.00539). The protein ITGB3 was detected in 10 out of 11 SqC samples at levels of 32.5 ± 7.9 nM, and only in 3 out of 23 LAC samples at levels of 11.9 ± 10.9 nM. Finally, the protein PACSIN2 was found in six LAC samples and in three SqC samples in almost equimolar concentrations of 2.1 ± 0.6 and 2.3 ± 0.3 nM, respectively (Figure 3d–f).

### 2.3. Proteomic Signature of EVs Distinguishes Lung Adenocarcinoma and Squamous Cell Carcinoma Histological Types, and Its Components Are Involved in Focal Adhesion

**Figure 4:**
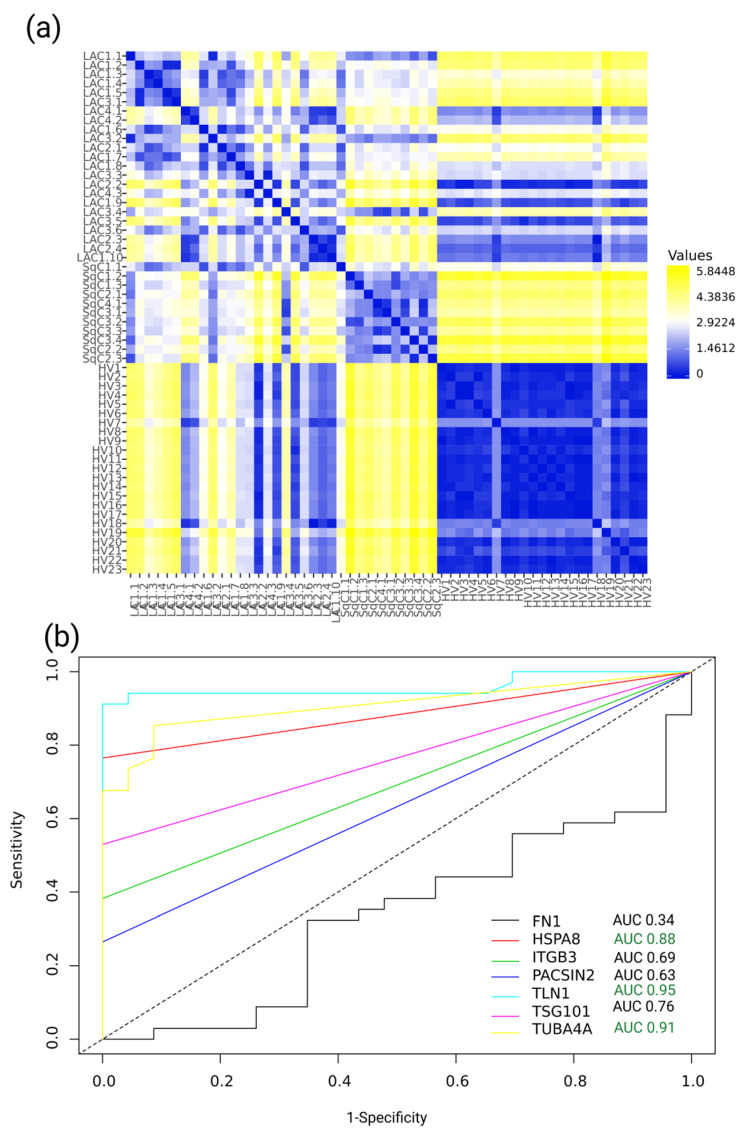
(**a**) Distance matrix of the experimental sample similarity based on the expression of components of the EV proteomic signature: FN1, TLN1, TUBA4A, HSPA8, ITGB3, TSG101, and PASCIN2. Blue denotes a lower distance and higher similarity between samples. (**b**) ROC curves for the components of the EV proteomic signature. The X-axis represents specificity, and the Y-axis represents sensitivity. ROC curves for FN1 (AUC, 0.34; *p*-value = 2.8 × 10^−2^), HSPA8 (AUC, 0.88; *p*-value = 3.9 × 10^−25^), ITGB3 (AUC, 0.69; *p*-value = 6.2 × 10^6^), PACSIN2 (AUC, 0.63; *p*-value = 5.7 × 10^−4^), TLN1 (AUC, 0.95; *p*-value = 1.5 × 10^−57^), TSG101 (AUC, 0.76; *p*-value = 1.1 × 10^−9^), and TUBA4A (AUC, 0.91; *p*-value = 1.2 × 10^−30^), with 95% CIs (computed with Delong’s method). Data are shown for patients with lung adenocarcinoma (LAC, N = 23), patients with lung squamous cell carcinoma (SqC, N = 11), and healthy volunteers (HV, N = 23).

The multiplex nature of the SRM method allows for the simultaneous analysis of numerous protein analytes. On the basis of the expression pattern of components of the EV proteomic signature, the distance matrix that reflects the degree of similarity between experimental samples is shown in Figure 4a. 

Figure 4a shows that the EV proteomic signature clearly distinguishes between LC and control samples. Moreover, we observed significant differences between the LAC and SqC samples, which are different histological subtypes of LC. The correlation matrix in Figure S10 demonstrates that the protein levels of TLN1, TUBA4A, and HSPA8 were the most important for sample allocation. Contrarily, FN1 demonstrates a lower level of correlation with the other components of the EV proteomic signature. 

To assess the potential of the EV proteomic signature components for LC recognition, we constructed a receiver operating characteristic curve (ROC) and calculated the area under it (the AUC). The top three classifiers were TLN1 (AUC, 0.95; *p*-value = 1.5 × 10^−57^; sensitivity, 0.91; specificity, 1; optimal cut-off point, 5.8 nM), TUBA4A (AUC, 0.91; *p*-value = 1.2 × 10^−30^; sensitivity, 0.85; specificity, 0.91; optimal cut-off point, 0.9 nM), and HSPA8 (AUC, 0.88; *p*-value = 3.9 × 10^−25^; sensitivity, 0.76; specificity, 1; optimal cut-off point, 8.1 nM). 

There were no significant differences for the EV proteomic signature when comparing the early and late-stage patients. Furthermore, the distance matrix for the early stage (1, 1A, and 1B) patients and healthy controls showed two clusters of LC patients vs. healthy volunteers (Figure S11). The level of the best classifier, i.e., TLN1, was 19-fold (*p*-value < 0.01) higher in the samples from early stage LC (1, 1A, and 1B) patients compared to the control samples. Moreover, TUBA4A’s frequency of detection and protein levels were higher in the early stage LC samples (median levels, 10.9 nM; N = 13) than the control samples (median levels, 2.8 nM; N = 2). Finally, the HSPA8 protein was only detected and quantified in samples from the early stage LC patients (median levels, 33 nM; N = 11). Despite the significant differences, the small early stage LC patient sample size (N = 13) should be kept in mind when interpreting the results.

To elucidate the biological function of our EV proteomic signature, we performed a search for potential protein–protein interactions.

**Figure 5:**
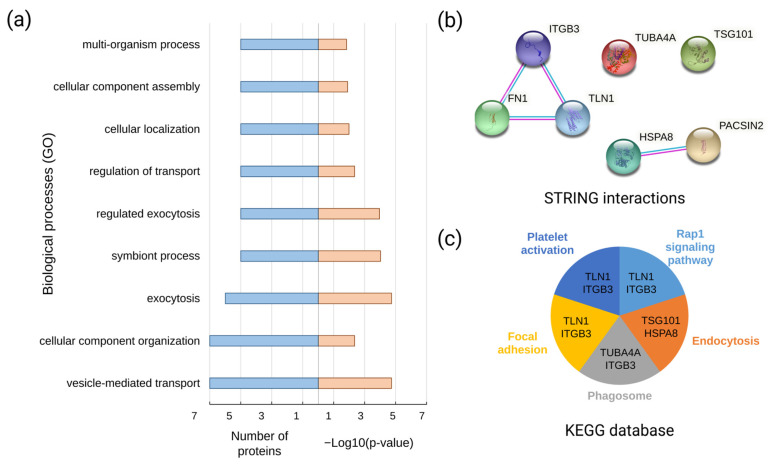
(**a**) The functional annotation of the EV proteomic signature components against categories of biological processes (Gene Ontology); (**b**) results of the STRING interaction analysis (PPI enrichment *p*-value: 0.0249) with the highest confidence (0.9) (the active interaction sources were experiments and curated databases); and (**c**) the functional annotation of the EV proteomic signature components against KEGG database pathways.

The STRING interaction analysis (Figure 5) revealed that the components of the EV proteomic signature detected in the blood plasma of LC patients (FN1, TLN1, TUBA4A, HSPA8, TSG101, ITGB3, and PACSIN2) were enriched in their interactions (PPI enrichment *p*-value: 0.0249) with the highest confidence (0.9), forming two potential complexes: FN1-ITGB3-TLN1 and HSPA8-PACSIN2. Functionally, the components of the EV proteomic signature were involved in focal adhesion (KEGG), the Rap1 signaling pathway (KEGG), and vesicle-mediated transport (biological processes, GO).

To interrogate the association of expression levels and patient survival, we used the UALCAN online platform and data on transcript expression levels for LC patients obtained from The Cancer Genome Atlas (TCGA). From seven components of EV proteomic signatures, high expression levels of TUBA4A and TSG101 were associated with poor survival (TUBA4A, *p*-value = 0.014 and TSG101, *p*-value = 0.039). Moreover, transcript levels of TUBA4A were significantly (*p*-value < 1 × 10^−12^) elevated in LC patients compared to healthy controls, whereas transcript levels of TSG101 were insignificantly altered (Figure S12).

## 3. Discussion

Extracellular vesicles released by cancer cells contain DNA, RNA, and proteins that reflect the molecular landscape of the producer cell [22]. On the other hand, EVs are not functionless, small “doppelgangers” of malignant cells; they play a role in numerous biological processes, including sending inhibitory or stimulatory growth signals to close and distant cells. EVs can affect the extracellular matrix (ECM) by modulating tumor immunity responses and can even transfer active oncogenes, e.g., EGFR or the mutant form of KRAS [23,24,25,26]. Thus, EVs represent a rich source of potential biomarkers that could contribute to the diagnosis of cancer and to the prognosis of outcomes (prognostic markers) and responses to treatment (predictive markers). Moreover, EVs have been found in various bodily fluids, including blood, urea, saliva, etc. [27]. Therefore, the analysis of the molecular composition of EVs is relevant to the field of liquid biopsy. 

Based on the results of our previous studies, we selected 23 EV-associated proteins and five convenient EV markers for validation using quantitative mass spectrometry with EV and WhL samples derived from A549 and NCI-H23 cells and with blood plasma obtained from LC patients and healthy volunteers. By applying SRM/SIS to the cell models, we detected 27 out of the 28 EV-associated proteins studied. The tetraspanin family member and one of the convenient EV markers, the CD82 protein, was not found in either the EV samples or WhL samples from the A549 and NCI-H23 cells. This can be explained by the fact that, according to the Protein Atlas database, the CD82 protein is not expressed in lung tissues.

For each cell line studied, the majority of proteins found in the WhL samples overlapped with the proteins detected in the EV samples, illustrating the molecular similarity of vesicles and producer cells. Furthermore, four proteins (MFGE8, ITGB3, HSPG2, and EPS15) were detected exclusively in the EV samples derived from both LC cell lines. Among them, epidermal growth factor receptor substrate 15 (EPS15) is involved in the internalization of ligand-inducible receptors, including EGFR [28]. Another EV-associated protein, PACSIN2, which was detected in LC patient blood plasma, is also involved in EGFR internalization [29]. EGFR’s aberrant expression and activation is associated with various types of cancer, including lung cancer. Notably, in the case of gastric cancer, EGFR can be delivered in tumor-derived exosomes into the liver where it fuses with the plasma membranes of liver stromal cells, preparing the metastatic niche [24]. It is possible that a similar process occurs in lung cancer, also involving the EGFR binding partners EPS15 and PACSIN2.

The targeted mass spectrometry analysis resulted in the detection of 7 (FN1, TLN1, TUBA4A, HSPA8, TSG101, ITGB3, and PACSIN2) out of 28 of the EV-associated proteins in the undepleted blood plasma of LC patients and healthy volunteers. Together, these proteins were denoted as an EV proteomic signature. Undepleted blood plasma represents a very complex biological matrix, with a protein concentration dynamic range exceeding 10 orders of magnitude [30]. The most abundant proteins, e.g., albumin, immunoglobulins, transferrin, etc., make up more than 90% of the total protein content, hampering the detection of lower abundance proteins by mass spectrometry. The removal of unwanted high-abundance proteins could enhance the detection sensitivity, but this would affect reproducibility. The highly selective and sensitive SRM/SIS method allows for the effective analysis of low-abundance proteins even without the removal of interfering highly abundant proteins [31].

Fibronectin, one of the components of the EV proteomic signature, is an extracellular matrix glycoprotein with aberrant expression in many types of cancer [32]. Fibronectin 1 (FN1) is FDA-cleared as a protein analyte for diagnostic tests. Increased production and deposition of FN1 dramatically changes the EMC’s properties at the onset of metastasis. However, this EV proteomic signature component failed to distinguish the LC samples from the healthy controls, or between different histological types of cancer. It should be mentioned that the FN1 protein-coding gene produces up to 17 distinct isoforms (according to the Uniprot database), which differ in terms of solubility, receptor-binding ability, spatiotemporal expression, and tissue localization. All the FN1 isoforms can be divided into two major classes: soluble plasma isoforms (pFN1) and insoluble cell isoforms (cFN1). Normally, pFN1 isoforms are secreted by hepatocytes into the blood circulation. Moreover, cFN1 isoforms are of high biological importance in relation to cancer. However, as a result of the high homology, it is not possible to select isoform-specific tryptic peptides for the majority of splice variants. The tryptic peptides that were used for the SRM analysis in the present study were mapped into 15 out of the 17 available splice variants. Consequently, the analysis provides data on the quantitative contents of the isoform mixture. Very few isoforms, e.g., isoform two (migration stimulation factor FN70), isoform five (fibronectin (V+I−10)-), isoform six (fibronectin (V+III−15)-), and isoform twelve, yield isoform-specific tryptic peptides. Furthermore, these peptides were only of a suitable length (9–20 amino acids) for SRM/SIS for two isoforms (two and five); i.e., they provide sufficiently high method selectivity and ionization efficacy. Notably, isoform two (migration stimulation factor FN70) was shown to be expressed by fetal and cancer patient fibroblasts [33]. In the future, assessing the levels of FN1 isoform two in LC patient blood with an isoform-specific peptide standard would contribute to the diagnostic power of the EV proteomic signature.

In contrast to the unchanged FN1 levels, six other components of the EV proteomic signature (TLN1, TUBA4A, HSPA8, ITGB3, TSG101, and PACSIN2) were upregulated in the LC samples, and TUBA4A, HSPA8, ITGB3, TSG101, and PACSIN2 were exclusively detected in the blood of LC patients. The most prominent marker, TLN1, is a key component of the focal adhesion complex. Global rearrangements of focal adhesion complexes accompany epithelial–mesenchymal transition (EMT), which plays an important role in metastasis [34]. Talin-1 also contributes to anoikin resistance, the mechanism by which cancer cells evade apoptosis after the loss of cell–cell adhesion and detachment from the ECM. Integrin beta 3 (ITGB3), another EV-associated protein detected in the LC samples, is involved in focal adhesion with TLN1 and is activated by the latter, which is in accordance with the results of the STRING analysis on protein–protein interactions. Previously, it was shown that talin levels were dramatically increased (> 16-fold) in highly metastatic cells as compared to cells with low metastatic potential [35]. In cell models of hepatocellular carcinoma, talin-1 inhibition or knockdown led to decreased proliferation, decreased migration, and enhanced anoikin effects, which suggests that the reverse of the EMT process is taking place [36]. 

The distribution of the TLN1 abundance or biological variability was much wider in the blood of LC patients than healthy controls. This suggests that, in cancer patients, elevated TLN1 levels indicate the presence of the disease and may correlate with the response to treatment or the severity of the prognosis. Notably, talin overexpression is associated with a poor prognosis for patients with different types of cancer, such as oral squamous cell carcinoma, nasopharyngeal carcinoma, and prostate as well as ovarian cancer [37,38,39,40]. However, to our knowledge, there is lack of data on lung cancer.

Along with TLN1, tubulin alpha-4A chain (TUBA4A) levels effectively distinguish LC patients from healthy controls. This protein is one of the tubulin alpha polypeptides and represents the constituent of cell microtubules. According to the Protein Atlas expression data, the majority of cancer tissues, and the majority of normal tissues, show moderate-to-strong cytoplasmic positivity. However, there is a lack of data on the role of this protein in oncogenesis.

Heat shock cognate 71 kDa protein (HSPA8 and HSC70) is involved in the synthesis, folding, transport, and degradation of proteins. That, in turn, affects the cell’s stress level and its survival. Considering the importance of cell proteostasis for oncogenesis, an upregulated abundance of HSPA8 is often associated with many cancer types [41]. Notably, high levels of HSPA8 were considered as a potential biomarker for endometrial cancer [42] and as a prognostic factor for lower overall survival in acute myeloid leukemia [43].

Intriguingly, it was shown that the levels of HSPA8 (HSC70) in the plasma measured by ELISA were dramatically decreased in lung cancer patients compared to healthy controls [44]. Contrarily, using mass spectrometry, we observed significant increases in HSPA8 levels in the blood plasma of LC patients. However, this controversial result may indicate the presence of abundant post-translational modifications (PTMs), e.g., phosphorylation, acetylation, ubiquitination, AMPylation, and ADP-ribosylation. Leaving the ternary and quaternary protein structure intact is pivotal for the success of the ELISA. Moreover, PTMs not only promote activation, inhibition, cleavage, degradation, etc., but also affect protein folding, which leads to the decreased affinity of monoclonal antibodies used in the ELISA. Furthermore, HSPA8 is a conventional marker of EVs, including exosomes. It is possible that the membranes that encapsulate the EV content protect the interaction between antibodies and HSPA8, and the harsh conditions involved in proteomic sample preparation (the high temperature, detergent usage, etc.) lead to the loss of lipid layer integrity and of EV proteins’ solubilization.

Tumor susceptibility gene 101 protein (TSG101) is a well-known EV marker involved in the biogenesis of exosomes, along with SDCBP, CD63, and syndecan. It is known that TSG101 interacts with ubiquitinated proteins and directs them into multivesicular endosomes. Aside from EV production, TSG101 is involved in recycling endosomes and cell cycle regulation. Formerly, TSG101 was considered to be an oncosuppressor [45]; however, thereafter, the content of this protein was found to be increased in various types of cancer, e.g., breast, ovarian, and hepatocellular carcinomas [46,47,48]. 

In our previous work, we determined FN1, TLN1, ITGB3, and TUBA4A to be “universal EV protein markers”. They are highly abundant in EVs derived from two cell lines of LC and three cell lines of colorectal cancer (CRC). Moreover, FN1 and TLN1 were designated core EV proteins based on the mass spectrometry profiling of vesicles from 60 different cell lines [49]. Furthermore, HSPA8 (HSC70) and TSG101 are known as conventional EV markers. In a recent study [17], PACSIN2 was found to be LC A549-cell-line-specific. Together, these observations suggest that FN1, TLN1, ITGB3, HSPA8, and TUBA4A detection in the blood using SRM/SIS may serve as an indicator of tumors, and elevations in their concentrations coupled with PACSIN2 detection discriminate lung cancer from other malignancies. Furthermore, bioinformatic analysis revealed that increased expression of TUBA4A and TSG101 was associated with a lower probability of patient survival, which indicates the prognostic value of the EV proteomic signature.

The expression profile for TLN1, TUBA4A, HSPA8, ITGB3, TSG101, and PACSIN2 was reasonably similar in the blood plasma of early and late-stage LC patients. Despite this, the EV proteomic signature allows one to distinguish between the blood plasma of early stage LC patients from that of healthy controls. Further studies focused on additional EV-associated proteins, which are characteristic of early stage LC, would expand the diagnostic capacity of the EV proteomic signature.

Despite the distinctive differences in the EV proteomic signatures of healthy volunteers and LC patients, it should be kept in mind that this signature may indicate the presence of chronic inflammation and may partially overlap with benign lung tumors and with tumors of different tissue origins. This should be explored in future experiments. Moreover, extended SRM/SIS experiments on a larger cohort of LC patients should be performed.

## 4. Materials and Methods

### 4.1. Cultivation of A549 and NCI-H23 Cell Lines and EV Isolation

The lung adenocarcinoma cell lines A549 and NCI-H23 were obtained from the cell culture bank of the Institute of Biomedical Chemistry (IBMC), Moscow, Russia. For proteomic analysis, the cells were cultured in a medium supplemented with exosome-depleted FBS. Cell lines were cultured until reaching 70–80% confluency in an atmosphere of 5% CO_2_ at 37 °C, in a DMEM/F-12 medium without glutamine (PanEco, Moscow, Russia), with the addition of 10% FBS (Thermo Fisher Scientific, Waltham, MA, USA), 1% GlutaMAX (Thermo Fisher Scientific, Waltham, MA, USA), 1% essential amino acids (NEAA, Thermo Fisher Scientific, Waltham, MA, USA), and 1% antimycotic antibiotics (amphotericin B 0.25 µg/mL, penicillin G 100 units/mL, and streptomycin 100 µg/mL). Cells were cultivated in 75 cm^2^ culture flasks with approximately 15 mL of medium. When the cells reached the monolayer (70–80% confluency, 20–30 × 10^6^ per flask), they were washed twice with phosphate-buffered saline (PBS) and the culture medium was replaced with an exosome-free medium (with the addition of FBS previously purified from exosomes via ultracentrifugation at 100,000× *g* for 14 h). For further analysis, the culture medium was collected after 24 h.

Isolation of EVs from the culture medium was performed as described earlier [17]. Briefly, the culture medium in an equal volume of 18 mL was centrifuged at 5000× *g* for 30 min at 4 °C (SX4750A-type rotor, Beckman Coulter, Allegra X-15R Centrifuge, Indianapolis, IN, USA) to remove cell debris. The resulting supernatant was passed through a 0.22 µm filter. After that, EVs were sedimented using an Optima MAX-XP Ultracentrifuge and a TLA-55 rotor (Beckman Coulter, Indianapolis, IN, USA) at 100,000× *g* (k-factor 123) for 120 min at 4 °C. The sediment was then resuspended in 50 µL of 0.015% sodium cholate in 0.1 M PBS, pH 7.4, and stirred with vertical rotation on a Bio RS-24 mini-rotator (Biosan SIA, Riga, Latvia) for 30 min at room temperature followed by ultracentrifugation under the conditions described above. The sediment obtained after the second ultracentrifugation was dissolved in 50 µL of 0.1 M PBS, pH 7.4, and layered on a 26% sucrose solution in a PBS (ρ = 1.1082 g/mL), followed by ultracentrifugation at 120,000× *g* (k-factor 102) for 120 min at 4 °C. The sediment was resuspended in 50 µL of 0.1 M PBS, pH 7.4, and frozen at −80 °C for subsequent proteomic analysis. For each cell line, exosome isolation was performed in three replicates.

### 4.2. Clinical Sample Description

Whole-plasma samples were obtained from 34 patients with non-small-cell lung cancer (NSCLC) (24 men and 10 women) aged from 46 to 77 years (mean age 61 ± 8.02 years). In 13 patients, stage 1 of lung cancer was diagnosed (including 1, 1A, and 1B), 7 patients had stage 2 lung cancer (including 2, 2A, and 2B), 10 patients had stage 3 lung cancer (including 3, 3A, and 3B), and in 4 patients stage 4 lung cancer was diagnosed. In the context of the international classification of lung cancer based on the TNM system, 18 patients had metastases to the regional lymph nodes (N1–N3), while 4 patients had distant metastases (M1). Histological examination of lung tissue samples showed that 23 patients had lung adenocarcinoma (LAC); these included 15 men and 8 women aged from 46 to 77 years old (mean age was 60 ± 8.16 years). In 11 patients squamous cell carcinoma of the lung (SqC) was diagnosed; these included 9 men and 2 women aged from 46 to 73 years (mean age 61.7 ± 8.06 years).

Plasma samples of 23 healthy volunteers (10 men and 13 women aged 23 to 42 years; median, 30 years) were used in this study. Venous blood was collected in vacuum tubes with K2 EDTA; plasma was obtained by centrifugation of whole blood at 1300 g for 10 min immediately after sampling. Hemolysis was assessed by visual inspection. The resultant plasma aliquots (200 μL) were stored at –80 °C until analysis.

### 4.3. Sample Preparation for Mass Spectrometry Analysis

For sample preparation prior to targeted mass spectrometry, plasma aliquots of 1.4–4.2 µL (average 2.6 µL, median 2.8 µL) containing 175 µg of total protein were used. Each blood plasma sample was subjected to one-step disulfide bond breaking (reduction and alkylation) in a 50 mM triethylammonium bicarbonate buffer (TEAB) (Sigma-Aldrich, St. Louis, MO, USA) (pH 8.5) containing 50 mM tris (2 carboxyethyl) phosphine (TCEP) (Thermo Fisher Scientific, Waltham, MA, USA) and 80 mM chloroacetamide (CAA) (Sigma-Aldrich, St. Louis, MO, USA) at 80 °C for 40 min. The reaction mixture was diluted with 100 µL of 50 mM TEAB (pH 8.5), and a Protease MAX (Promega, Fitchburg, WI, USA) was added to a final concentration of 0.1% along with the trypsin solution containing 2.5 µg of trypsin (Promega, Fitchburg, WI, USA), followed by incubation overnight at 37 °C. Hydrolysis was stopped by adding formic acid (Sigma-Aldrich, St. Louis, MO, USA) to a final concentration of 5%. In the obtained samples, the peptide concentrations were determined by a colorimetric method using a Pierce Quantitative Colorimetric Peptide Assay kit (Pierce, Rockford, IL, USA) in accordance with the manufacturer’s recommendations. Samples were dried using a SpeedVac vacuum concentrator (Thermo Scientific, Waltham, MA, USA) and resuspended in 0.1% FA solution containing SISs to a final content of 40 fmol for each SIS per µg of total peptides. EV and WhL samples were digested according to the protocol described previously [17].

### 4.4. Synthesis of SISs

For the identification and quantitative measurements of selected proteins, synthetic peptides were used as an internal standard. The amino acid sequences of these peptides were identical to their natural counterparts but contained an amino acid with the inclusion of stable isotopes (Lys or Arg ^13^C_6_,^15^N_4_). The physicochemical features of standard peptides and their natural counterparts are the same; therefore, they co-elute from the reverse phase column, but molecular mass differences are of 8 Da (heavy Lys (K)) or 10 Da (heavy Arg (R)). Knowing the concentrations of synthetic peptides, the abundance of natural counterpart can be calculated.

The target peptides were selected from the shotgun mass spectrometric data obtained in our previous experiment [17]. The criteria of selection were as follows: The amino acid sequence had to be unique within the biological species Homo sapiens. The amino acid sequence did not contain cysteine (C), N-terminal glutamic acid (E), or tryptophan (W), and was missing hydrolysis sites. The length of peptides had to be within the range of 9–20 amino acid residues. For proteins with a large number of peptides, the quality of the high-resolution MS spectra was manually evaluated.

Solid-phase peptide synthesis was performed using an Overture™ Robotic Peptide Library Synthesizer (Protein Technologies, Manchester, UK), as described previously [11]. In the synthesis of isotope-labeled peptides, the isotopically labeled amino acids Fmoc-Lys-OH-13C6.15N or Fmoc-Arg-OH-13C6.15N (Cambridge Isotope Laboratories, Cambridge, MA, USA) were used instead of the usual lysine or arginine.

### 4.5. Quantitative Analysis of EV-Associated Proteins by Targeted Mass Spectrometry

For SRM/SIS analysis 14.5 µg of total peptide was used for each sample per LC-SRM run. Each experimental sample was analyzed in three technical replicates. Before analysis, the samples were dried in a vacuum concentrator and reconstituted in 0.1% formic acid containing SIS in an equimolar concentration of 500 fmol/µL. The final content of each SIS was 40 fmol/µg of total peptides.

Chromatographic separation was performed using an Agilent 1200 series system (Agilent Technologies, Santa Clara, CA, USA) connected to a TSQ Quantiva triple quadrupole mass analyzer (Thermo Scientific, Waltham, MA, USA). A sample was separated using an ZORBAX SB-C18 analytical column (150 × 0.5 mm, 5 μm particle diameter) (Agilent Technologies, Santa Clara, CA, USA) in a gradient of acetonitrile with a flow rate of 20 μL/min. First, the column was equilibrated with 5% solution B (80% acetonitrile in 0.1% formic acid) and 95% solution A (0.1% formic acid) for 5 min. Then, the concentration of solution B was linearly increased to 50% for 30 min, after which the concentration of solution B was increased to 99% in 1 min, and the column was washed with 99% solution B for 5 min. Then, the concentration was returned to the initial conditions for 1 min, in which the column was balanced for 9 min. A mass spectrometry analysis was performed in the dynamic selected reaction monitoring (dSRM) mode using the following settings of the MS detector: The capillary voltage was 4000 V, the velocity of the drying gas (nitrogen) was 7 L/min, the velocity of the axillary gas (nitrogen) was 5 L/min, the capillary temperature was 350 °C, the isolation window for the first and third quadrupole was 0.7 Da, the scan cycle time was 1.2 s, and the collision gas (argon) pressure in the second quadrupole was set at 1.5 mTorr. The retention time window on the reverse phase column was 2.2 min for each precursor ion. The transition and normalized collision energy (V) lists are presented in Supplementary Table S1 (“SRM Table”).

The results were analyzed and plotted using Skyline MacCoss Lab Software (version 4.1.0) to compare chromatographic profiles of the endogenous peptide and the corresponding SIS standard. The peak area ratio for the endogenous peptide and the corresponding SIS standard was automatically calculated in Skyline. To determine the amount of protein, the ratio calculated in Skyline was multiplied by the known content of each SIS standard. The measurement of each EV-associated protein was taken as the mean value of the content calculated from the results of SRM analysis in triplicate for a plasma sample after tryptic digestion, performed in a single replicate. The target protein content was expressed in fmol per μg of total protein, and then converted to molar concentration in nmol/L (nM).

### 4.6. Statistical and Bioinformatic Analysis

The receiver operating characteristic (ROC) curve was obtained using easyROC: a web tool for ROC curve analysis (ver. 1.3.1) web app [50]. The Youden index was applied to dichotomize the cut-off point. Sample sizes were obtained at the levels of 21 (control) and 21 (case), calculated with single test (type I error = 0.01, power 0.9, AUC 0.8, and allocation ratio 1).

The box plots were obtained using BoxPlotR: a web tool for generation of box plots. 

The STRING database v.11.0 was used to retrieve the protein–protein interactions (PPIs) between components of the EV proteomic signature detected in blood plasma of LC patients and/or healthy volunteers. A high confidence (0.9) score was applied. The active interaction sources were experiments and curated databases.

To interrogate the association of expression levels and patient survival, we used the UALCAN online platform (http://ualcan.path.uab.edu, accessed on 6 October 2021) and data on transcript expression levels for LC patients obtained from The Cancer Genome Atlas (TCGA).

## 5. Conclusions

In the current study, we detected several proteins (FN1, TLN1, TUBA4A, HSPA8, ITGB3, TSG101, and PACSIN2) which we denoted as an EV proteomic signature in the blood plasma of LC patients, taking advantage of the selectivity, sensitivity, and multiplicity of the SRM/SIS method. The expression pattern of the EV proteomic signature effectively distinguished between LC patient samples and samples from healthy volunteers, and between the lung adenocarcinoma and squamous cell carcinoma histological types. The EV proteomic signature has the potential for use in early stage LC recognition and for prognosis of outcomes. The determined EV proteomic signature is flexible and could be expanded with additional protein components in a cost-effective manner if needed.

## Figures and Tables

**Table 1:** Patient and healthy volunteer characteristics. LAC—lung adenocarcinoma, SqC—lung squamous cell carcinoma, and HV—healthy volunteers.

	LAC	SqC	HV
Total	23	11	23
Age	46–77	46–73	23–42
Male	15	9	10
Female	8	2	13
Stage 1, 1A, and 1B	10	3	-
Stage 2, 2A, and 2B	4	3	-
Stage 3, 3A, and 3B	6	4	-
Stage 4	3	1	-

## Data Availability

The targeted mass spectrometric data have been uploaded to the PASSEL repository (dataset PASS01696).

## Supplementary Materials

The following are available online, Figure S1: Unicity checker Nextprot analysis of 36 prototypic peptides that were used for the measurement of the abundance of 28 EV-associated proteins. Figure S2: Correlation between measurements of FN1 levels in blood plasma (nM) performed by selected reaction monitoring (SRM) using proteotypic unique peptides STTPDITGYR and SYTITGLQPGTDYK as standards, and a histogram of the distribution of the coefficient of variation (CV). Figure S3-S9: High-resolution annotated MS2 spectrum of FN1-, TLN1-, TUBA4A-, TSG101-, ITGB3-, and PACSIN2-specific peptides. Figure S10: Correlation matrix for the abundance of components of the EV proteomic signature (FN1, TLN1, TUBA4A, HSPA8, TSG101, ITGB3, and PACSIN2). Figure S11: Distant matrix of experimental sample similarity based on the expression of components of the EV proteomic signature (FN1, TLN1, TUBA4A, HSPA8, ITGB3, TSG101, and PASCIN2). Figure S12: The association of TUBA4A and TSG10 expression levels and LC patient survival calculated in UALCAN platform. Table S1: List of EV-associated proteins and unique peptides; list of transitions (pairs of precursor ion–fragment ion) used for SRM/SIS analysis; patient information; and measurements of EV-associated proteins in undepleted blood plasma from lung cancer patients and healthy volunteers.

## Sample Availability

Samples of the SIS compounds are available from the authors.
