# Supplementary material for: Proteomic Signature of Extracellular Vesicles for Lung Cancer Recognition
Source: Molecules. 2021 Oct 12;26(20):6145. doi: 10.3390/molecules26206145 (PMC8539600; doi:10.3390/molecules26206145)
Supplement: Supplementary file 1 [file molecules-26-06145-s001.zip › molecules-1381069-supplementary.pdf]

molecules

MDPI

Article

Proteomic signature of extracellular vesicles for lung cancer recognition

Svetlana E. Novikova [1,†], Natalia A. Soloveva [1,*,†], Tatiana E. Farafonova [1], Olga V. Tikhonova [1], Pao Chi Liao [2] and Victor G. Zgoda [1,*]

1 Orekhovich Institute of Biomedical Chemistry, Russian Academy of Medical Sciences, Pogodinskaya 10, Moscow119121, Russia; novikova.s.e3101@gmail.com (S.E.N.); farafonova.tatiana@gmail.com (T.E.F.); tiolika@gmail.com (O.V.T.)
2 Department of Environmental and Occupational Health, College of Medicine, National Cheng-Kung University, 1 Dasyue Rd., East District, Tainan 701, Taiwan; liaopc@mail.ncku.edu.tw
* Correspondence: n.shushkova@yandex.ru (N.A.S.); victor.zgoda@gmail.com (V.G.Z.)
† These authors contributed equally to this work.

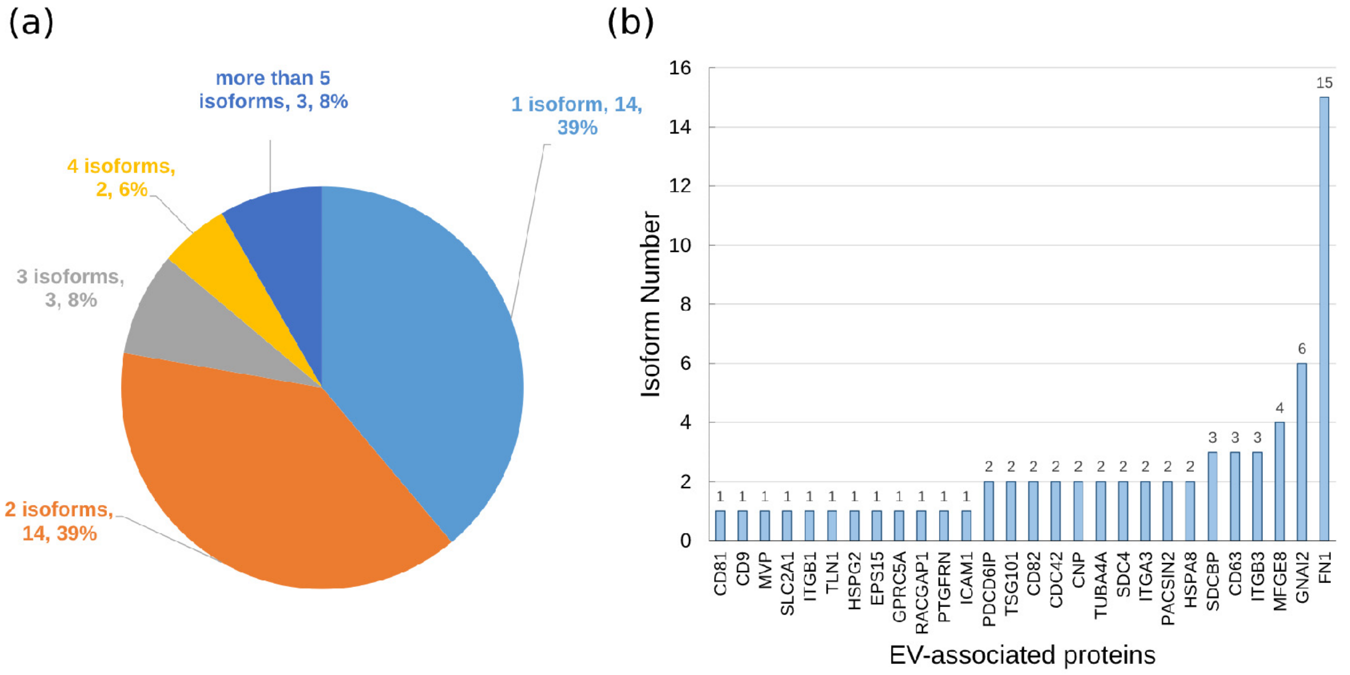

Figure S1. Unicity checker Nextprot analysis of 36 prototypic peptides that were used for measurement of abundance of 28 EV-associated proteins. (a) Distribution of isoform-specificity; (b) Isoform numbers that could be measured by surrogate peptide standard per each EV-associated protein (polypeptide product of one protein-coding gene).

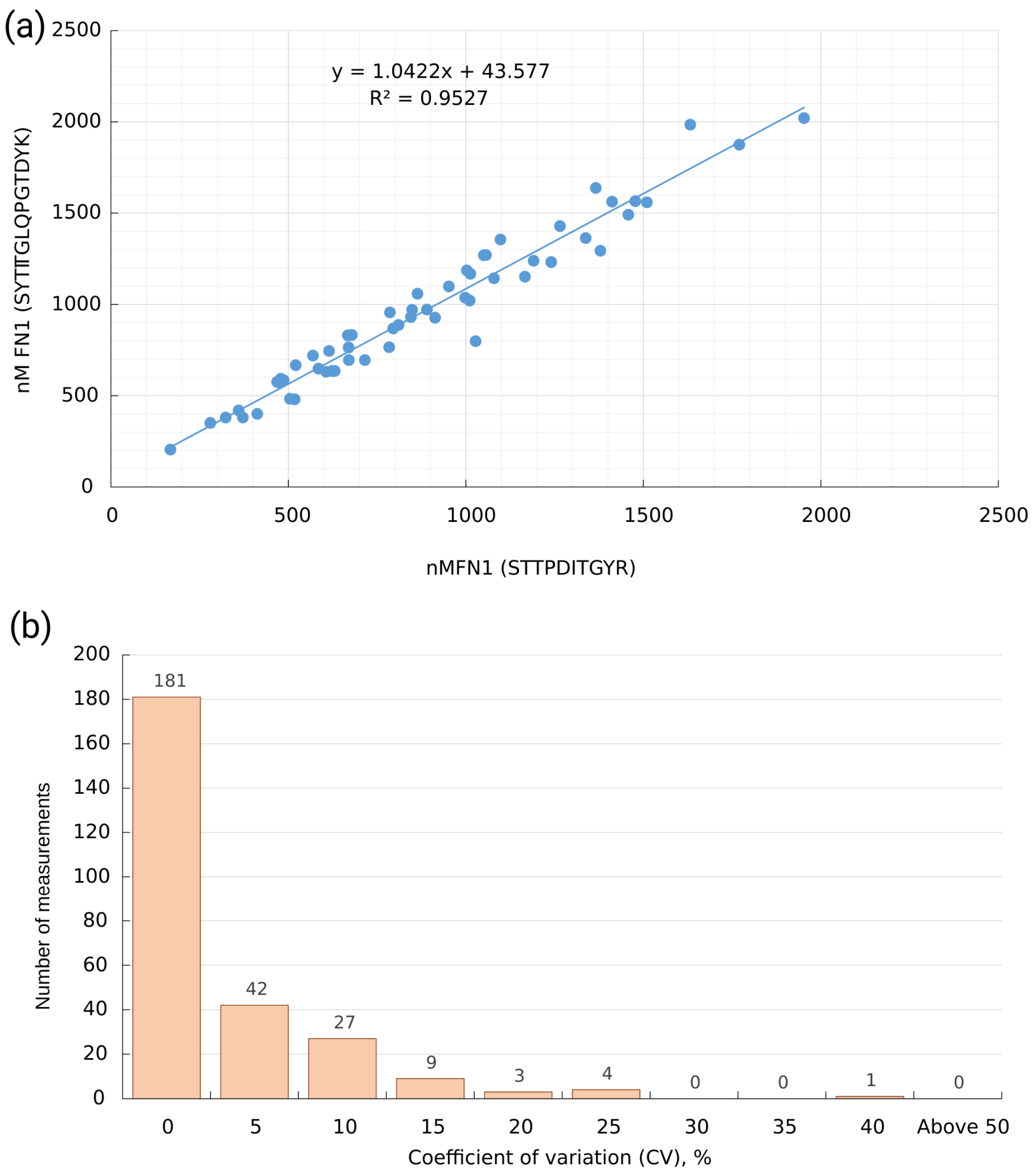

Figure S2. (a)Correlation between measurements of FN1 levels in blood plasma (nM) performed by selected reaction monitoring (SRM) using proteotypic unique peptides STTPDITGYR and SYTITGLQPGTDYK as a standards. (b) Histogram of distribution of coefficient of variation (CV) calculated for each measurement (N=267). The measurement is averaged value from 3 technical LC-SRM replicates.

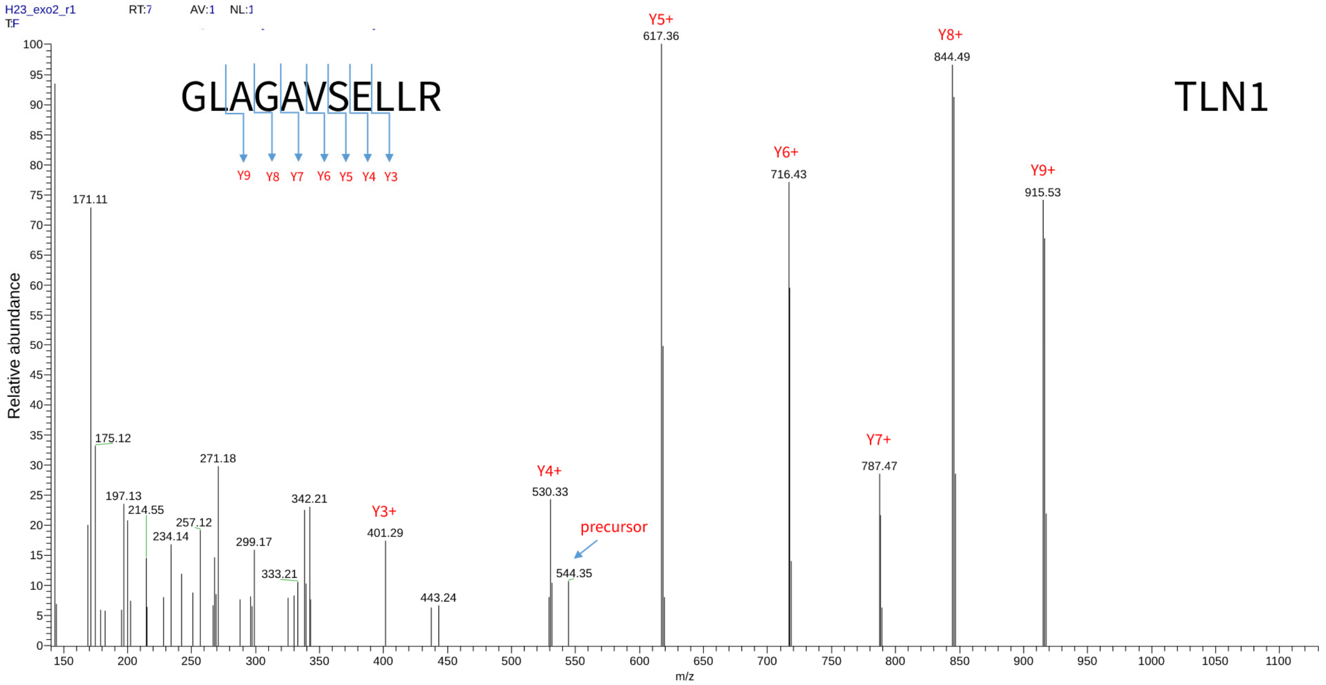

Figure S3. High-resolution annotated MS2 spectrum of TLN1-specific peptide GLAGAVSELLR.

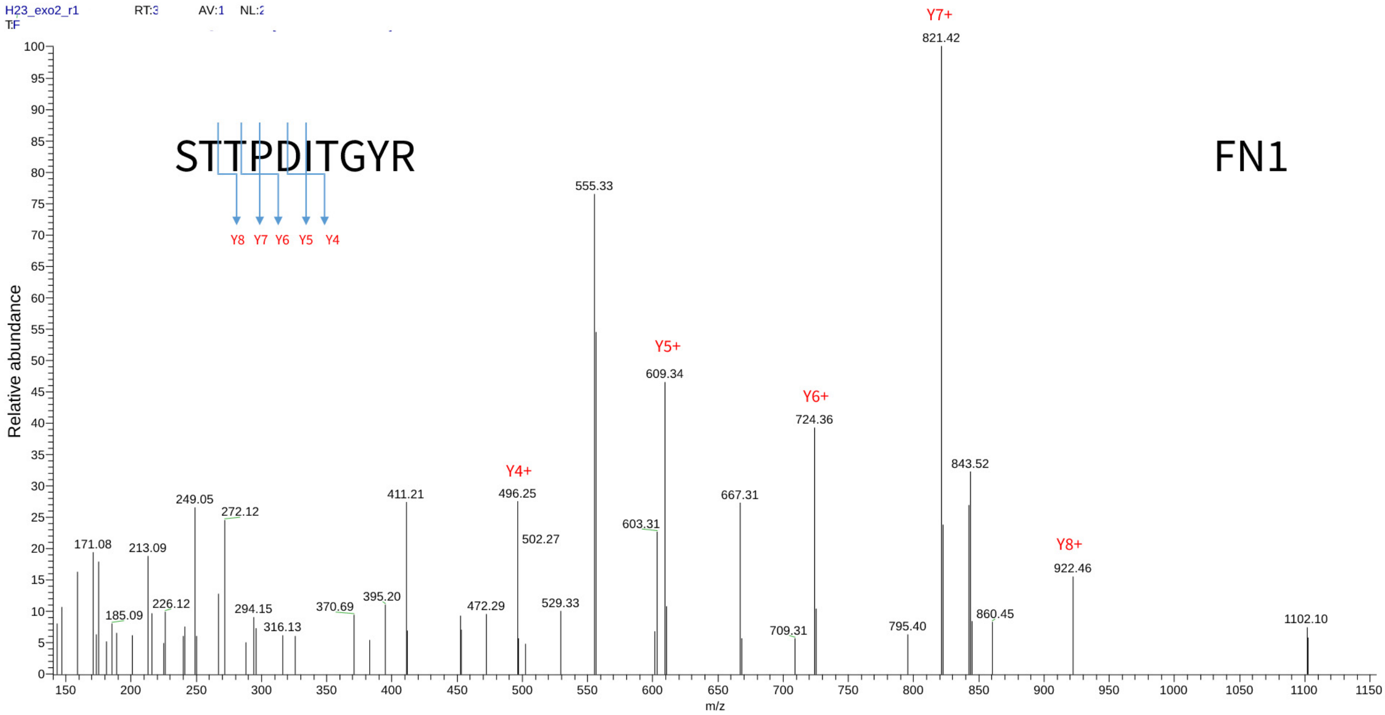

Figure S4. High-resolution annotated MS2 spectrum of FN1-specific peptide STTPDITGYR.

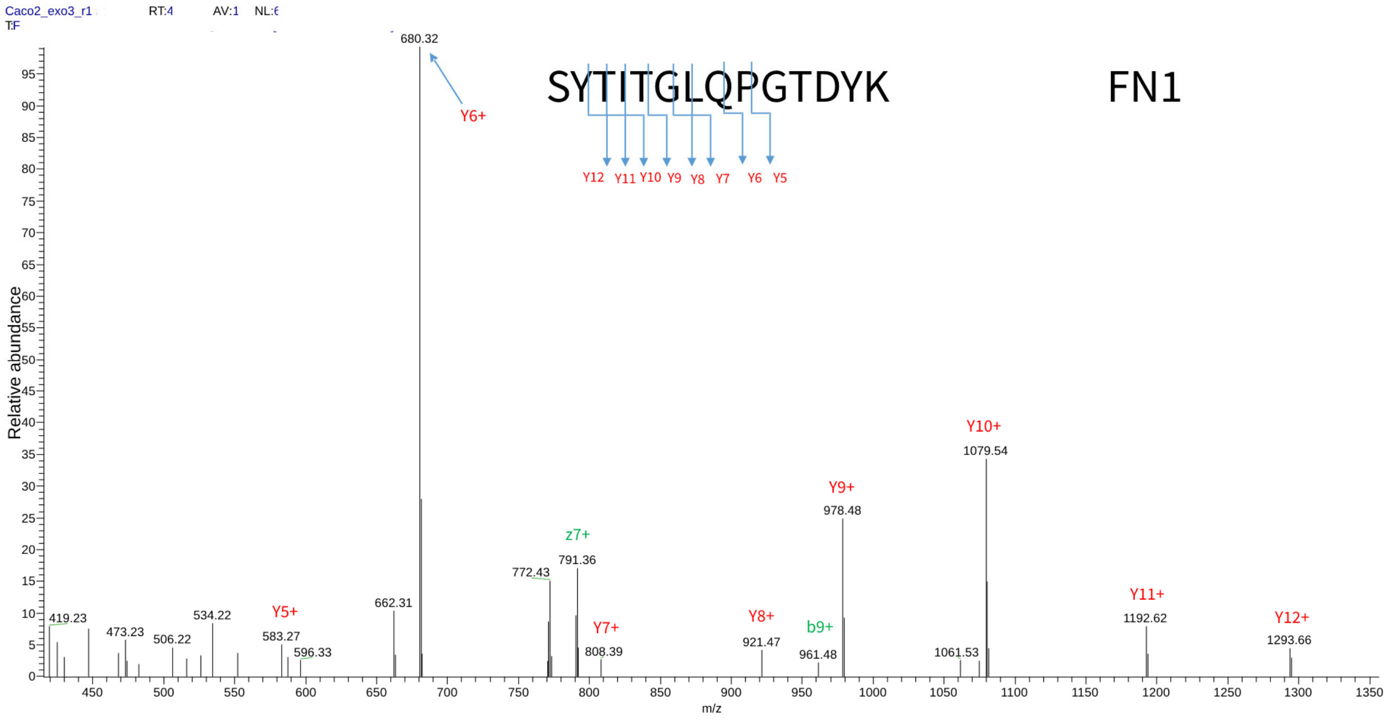

Figure S5. High-resolution annotated MS2 spectrum of FN1-specific peptide SYTITGLQPGTDYK.

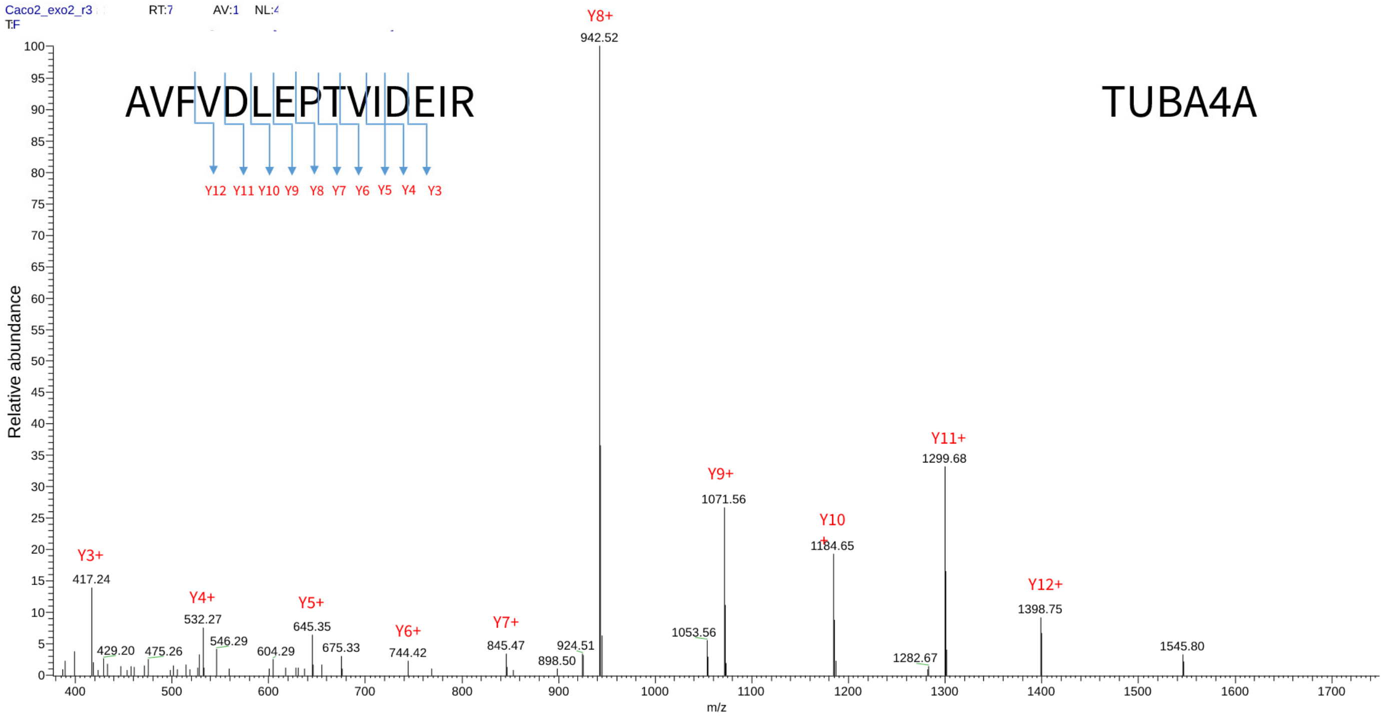

Figure S6. High-resolution annotated MS2 spectrum of TUBA4A-specific peptide AVFVDLEPTVIDEIR.

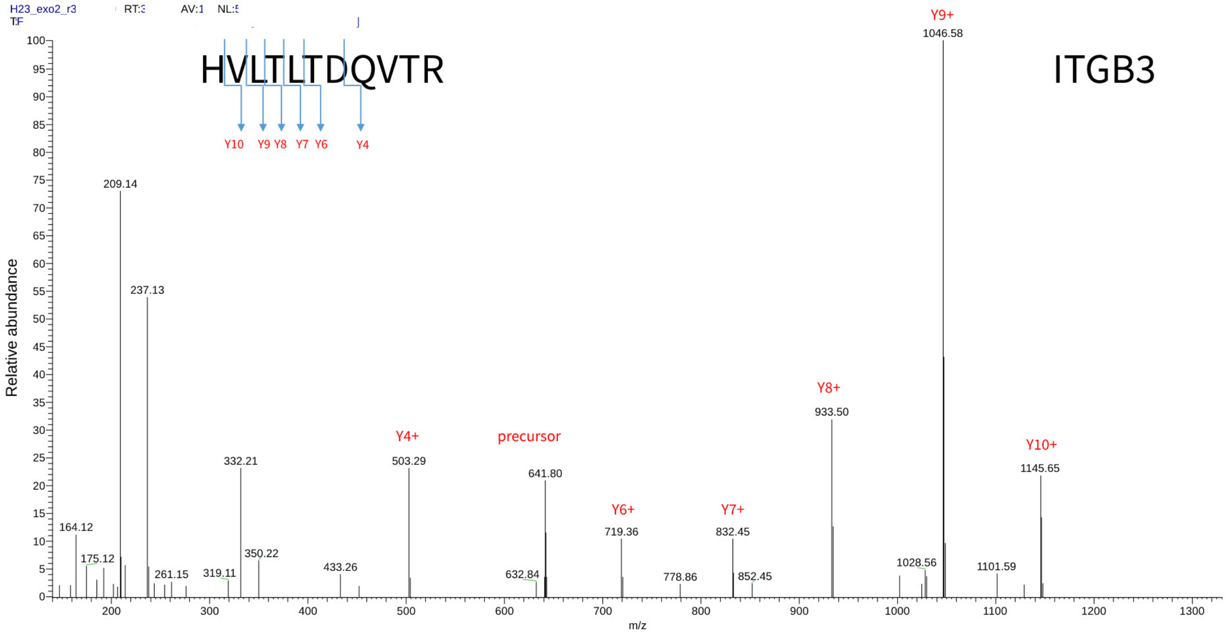

Figure S7. High-resolution annotated MS2 spectrum of ITGB3-specific peptide HVLTLTDQVTR.

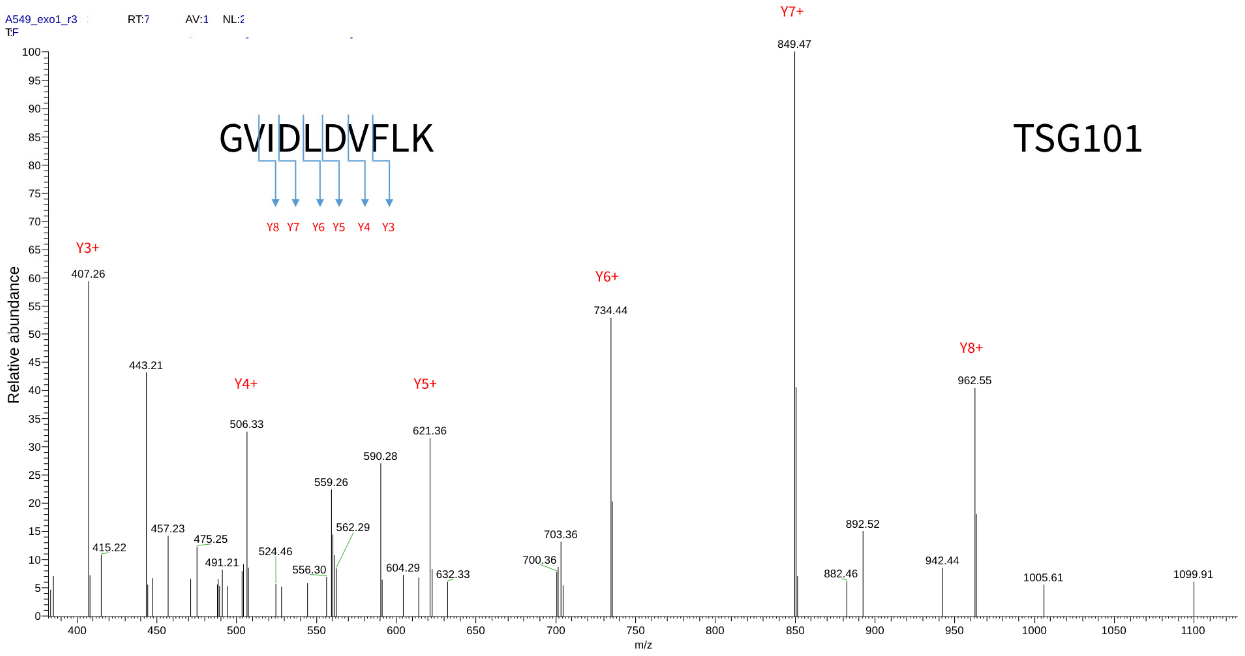

Figure S8. High-resolution annotated MS2 spectrum of TSG101-specific peptide GVIDLDVFLK.

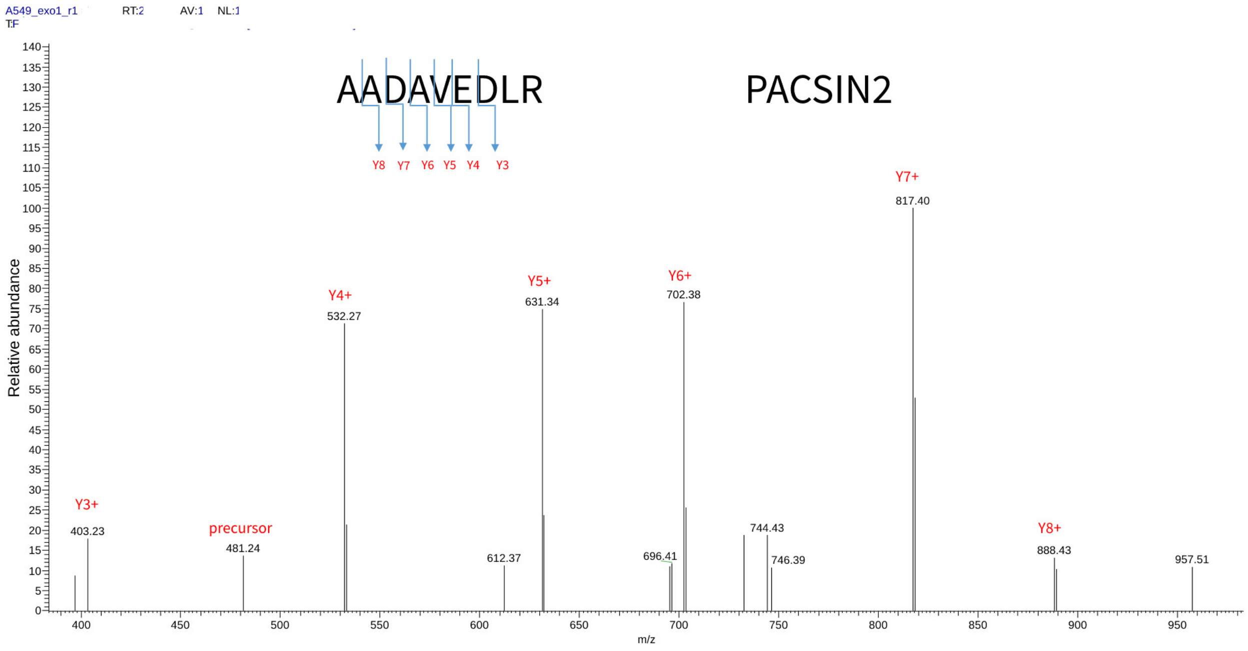

Figure S9. High-resolution annotated MS2 spectrum of PACSIN2-specific peptide AADAVEDLR.

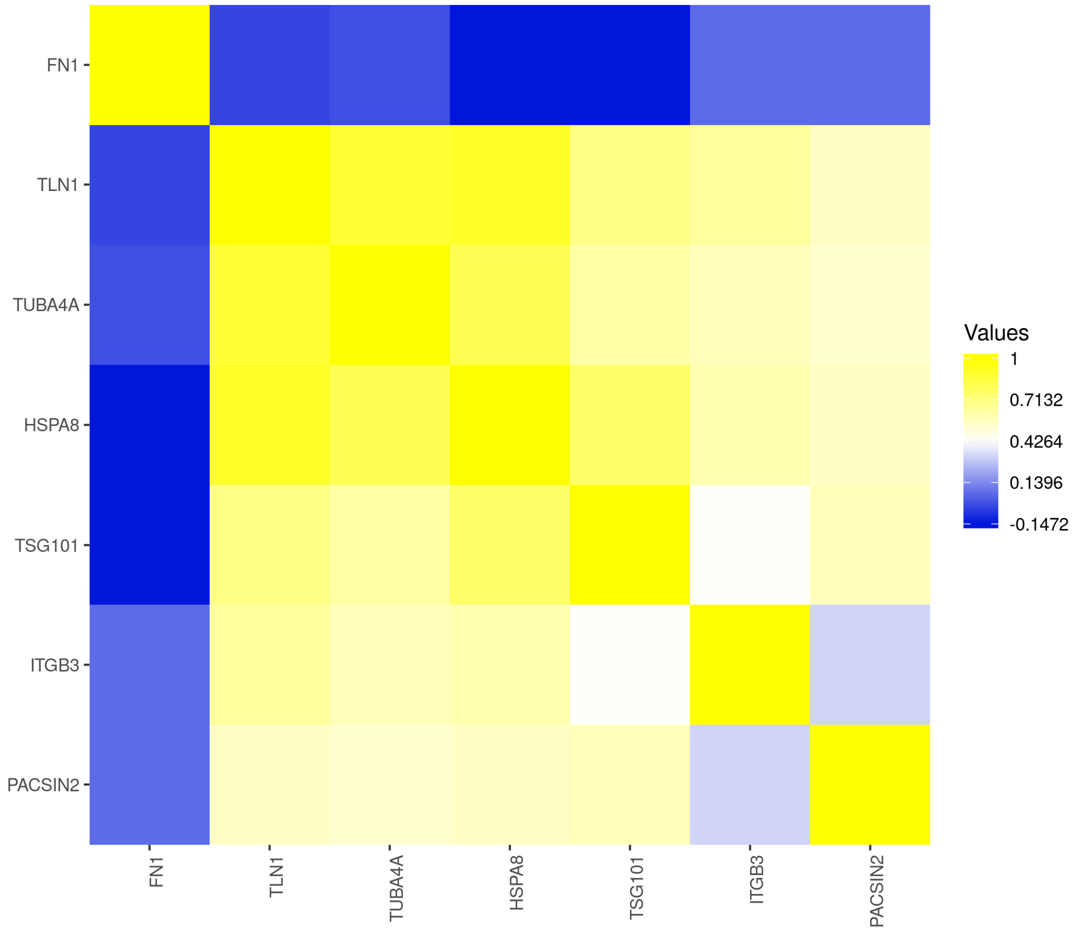

Figure S10. Correlation matrix for abundance of components of EV proteomic signature (FN1, TLN1, TUBA4A, HSPA8, TSG101, ITGB3, and PACSIN2) that were measured in blood plasma obtained from 23 patients with lung adenocarcinoma (LAC), 11 patients with lung squamous cell carcinoma (SqC), and 23 healthy volunteers (HK). The color (from yellow to blue) and its intensity reflect correlation in protein levels.

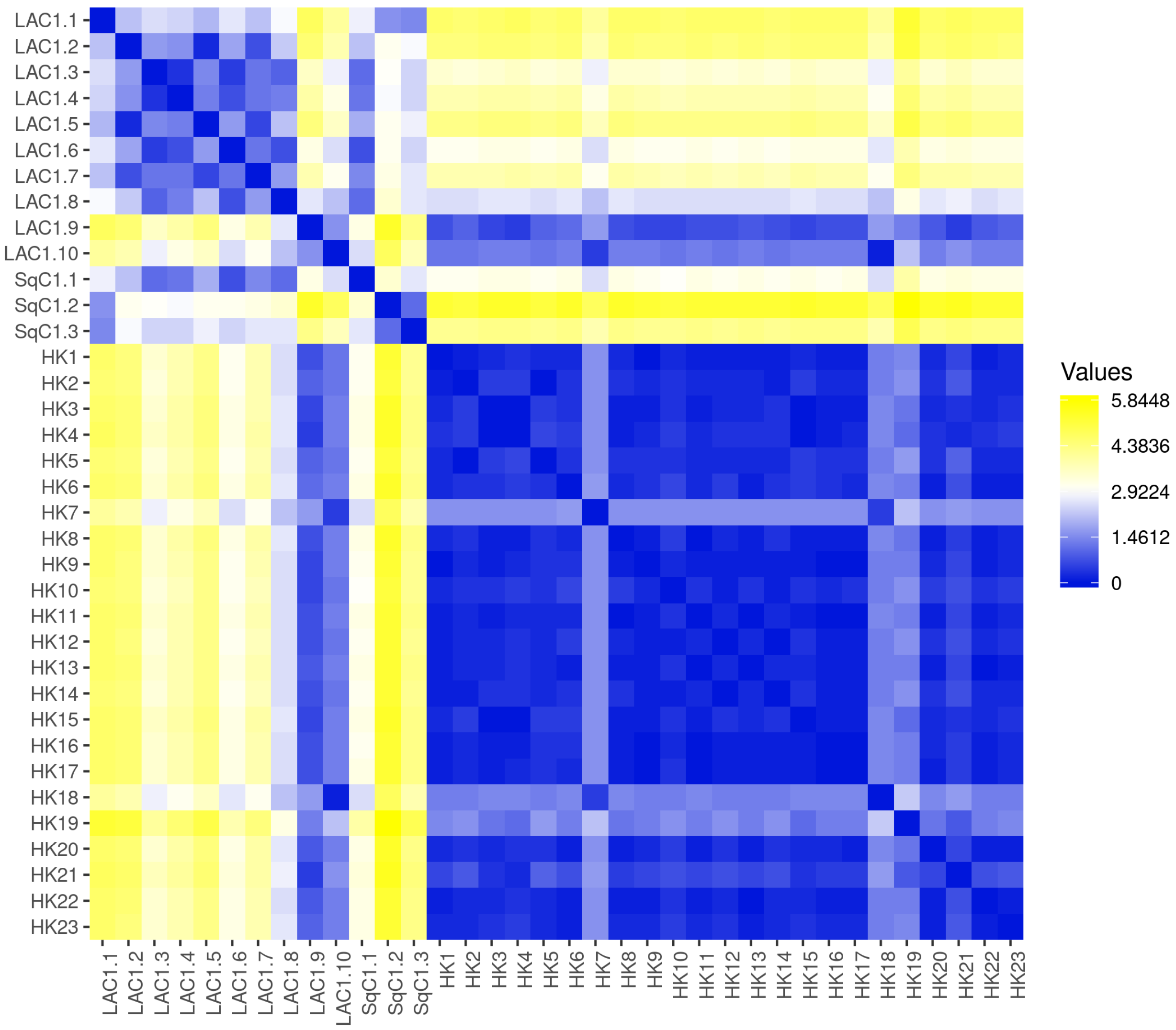

Figure S11. Distant matrix of experimental samples similarity based on expression of components of EV proteomic signature FN1, TLN1, TUBA4A, HSPA8, ITGB3, TSG101, and PASCIN2.). Data is shown for patients with early stage (1, 1A, and 1B) lung adenocarcinoma (N = 13) and healthy volunteers (HK, N =23).

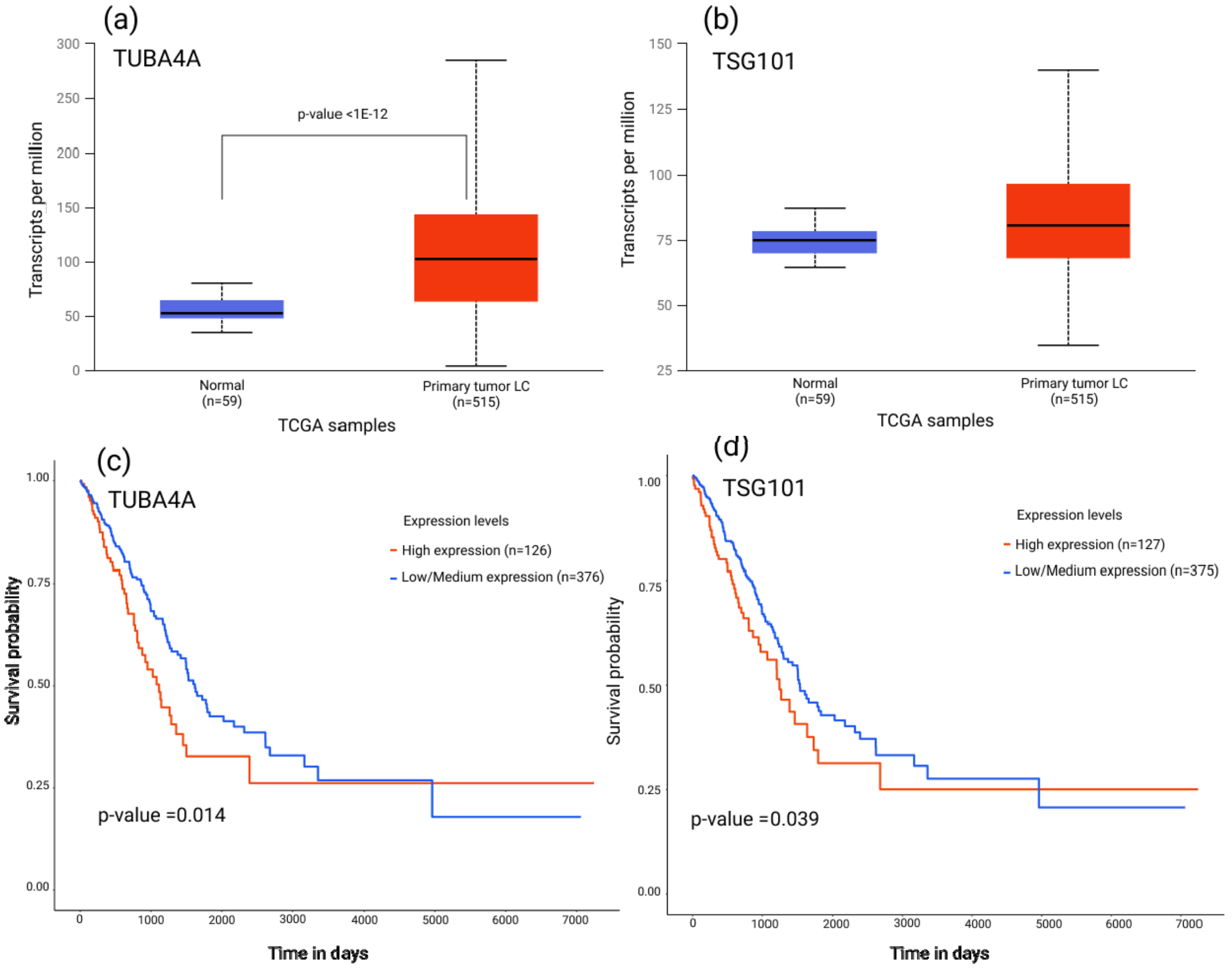

Figure S12. The association of TUBA4A and TSG10 expression levels and LC patient survival calculated in UALCAN platform. TUBA4A (a) and TSG101 (b) transcript expression levels for LC patients obtained from The Cancer Genome Atlas (TCGA). The transcript expression levels is shown in targeted transcripts per million of RNA molecules in the RNA-seq sample. Survival probabilities associated with high or low/medium TUBA4A (c) and TSG10 (d) expression.
